# Supplementary material for: DOK7 gene therapy enhances motor activity and life span in ALS model mice
Source: EMBO Mol Med. 2017 May 10;9(7):880–9. doi: 10.15252/emmm.201607298 (PMC5494517; doi:10.15252/emmm.201607298)
Supplement: Supplementary file 1 — Expanded View Figures PDF [file EMMM-9-880-s001.pdf]

Expanded View Figures

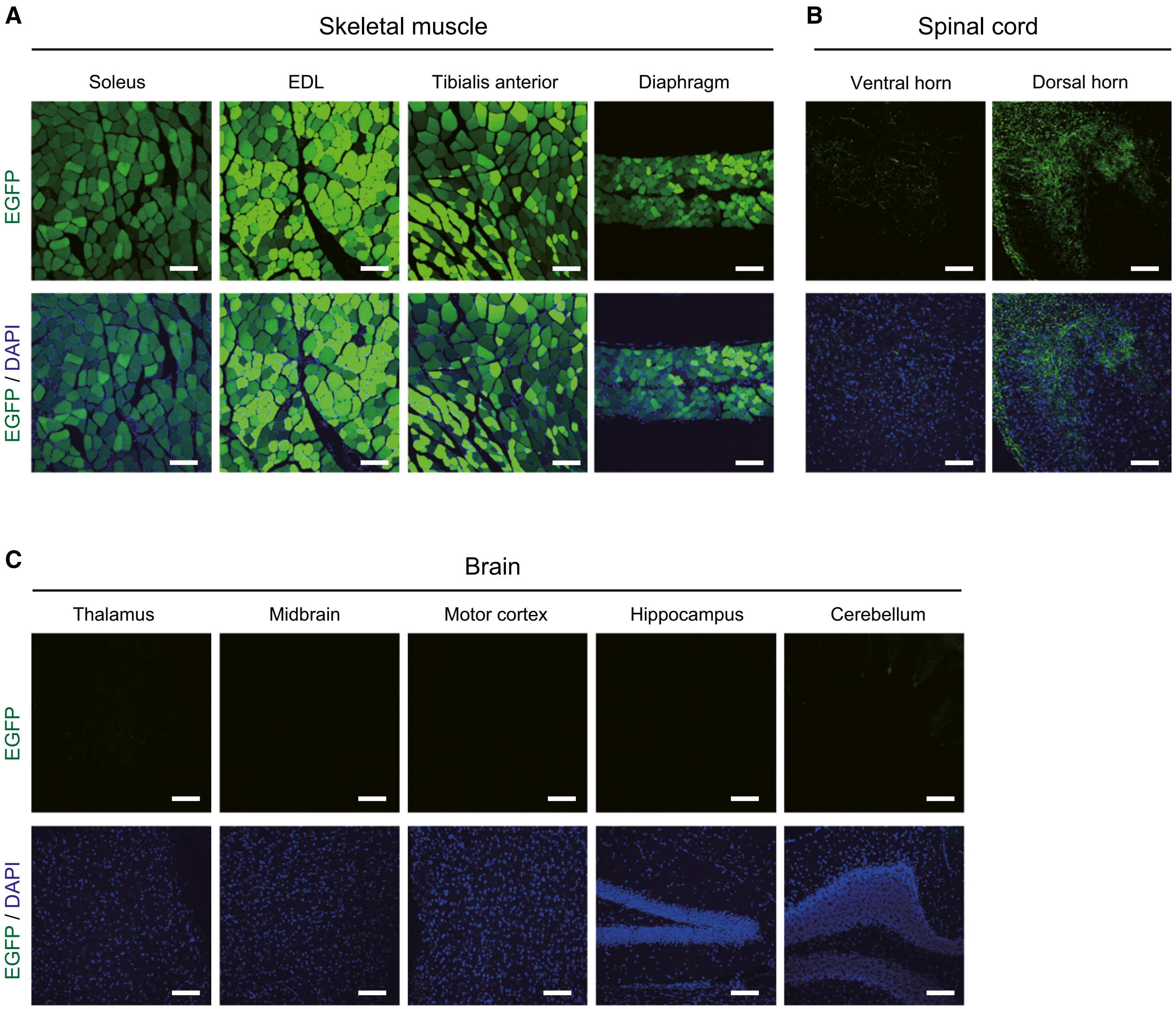

Figure EV1. Transduction of intravenously injected AAV-D7 is prominent in muscle, but only weakly and faintly detectable in spinal cord and brain, respectively, in ALS mice.

A–C ALS mice treated with 1.2×10^{12} vg of AAV-D7 at P90 were analyzed at P120. The expression of Dok-7-EGFP protein was monitored by EGFP fluorescence (green) and nuclei were visualized by DAPI (blue). High transduction was observed in the indicated skeletal muscles (A), but weak or faint transduction was detectable in the ventral (faint) and dorsal (weak) horns of the spinal cord (B) and in the cerebellum (faint) (C). Scale bars, 100 μm. EDL, extensor digitorum longus.

Figure EV2. *DOK7* gene therapy shows no obvious effects on motor neuron survival and motor axon atrophy in ALS mice.
WT or ALS mice treated or not with 1.2×10^{12} vg of AAV-D7 at P90 were analyzed at P120.

A Nissl staining of the L4-L5 spinal segment. Scale bars, 200 μm. Insets show higher magnification of the ventral horn in the boxed regions (scale bars, 100 μm). NT, not treated.
B Quantification of surviving motor neurons (n = 5 mice). Values are means ± SEM. ***$P < 0.0001$ by one-way ANOVA with Bonferroni's *post hoc* test. n.s., not significant.
C Anti-neurofilament-H staining of the L4 ventral roots. Insets show higher magnification of the boxed regions. Scale bars, 50 μm.
D Size distribution of motor axons (n = 5 mice). Values are means ± SEM.

© 2017 The Authors

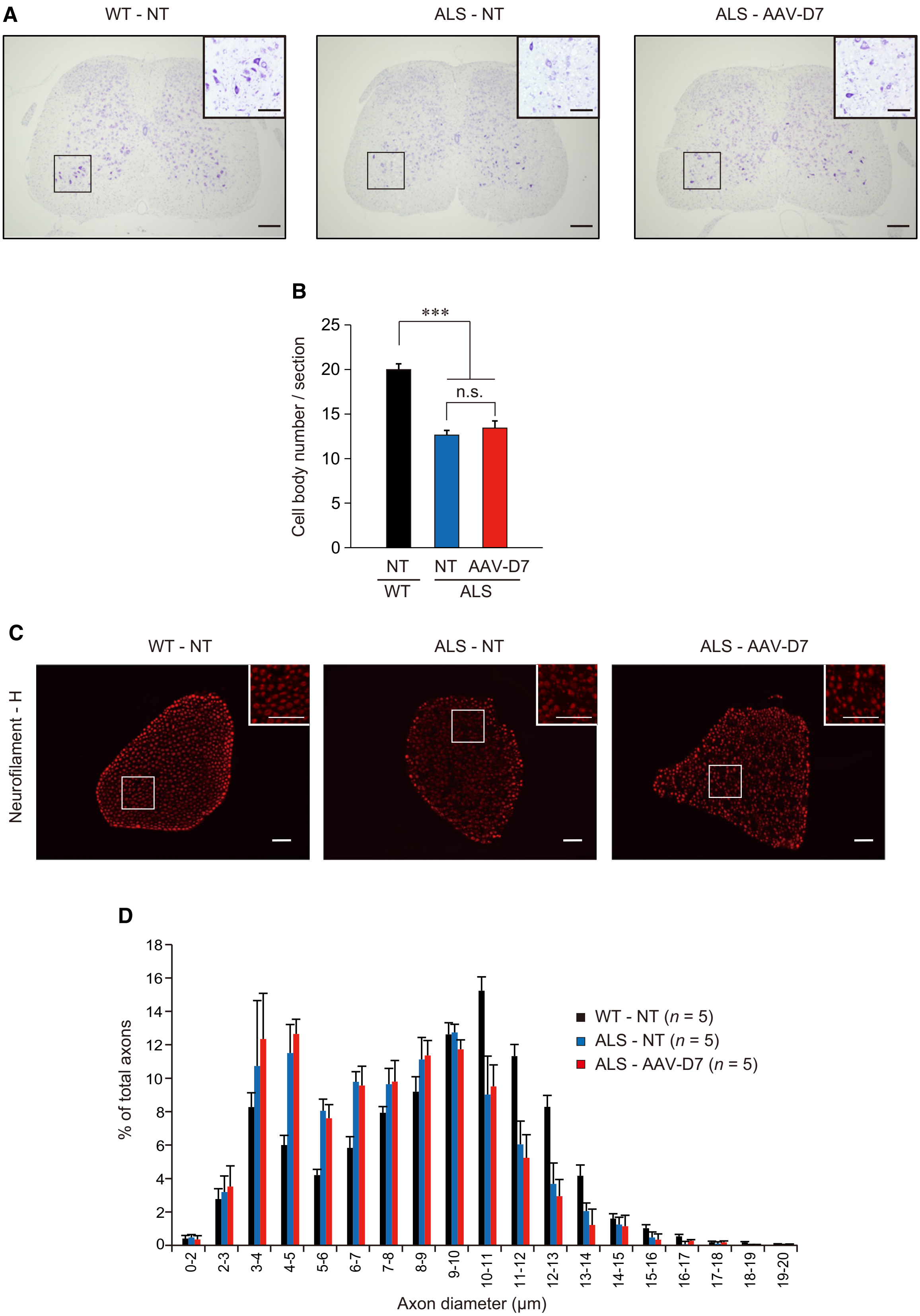

Figure EV2.

© 2017 The Authors

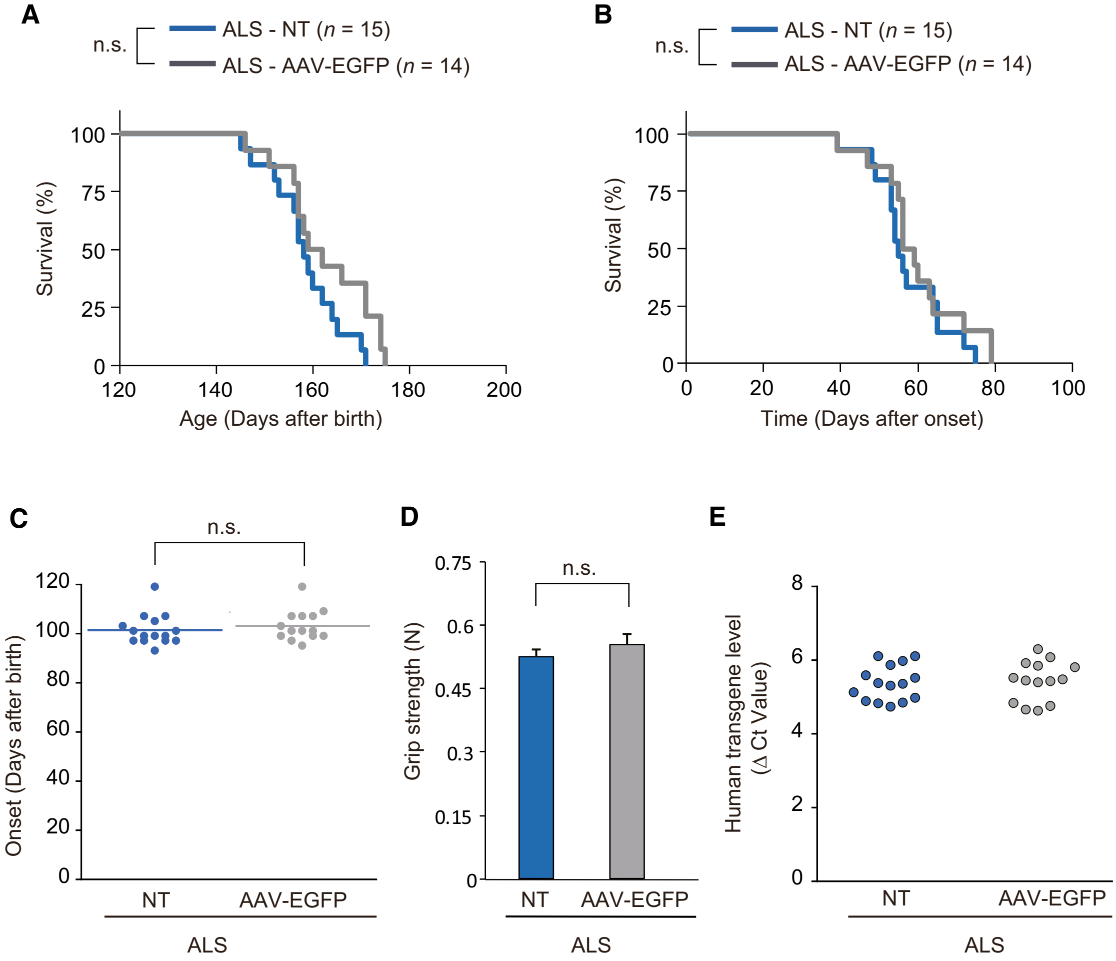

Figure EV3. Treatment with AAV-EGFP does not prolong survival in ALS mice.
ALS mice were treated or not with 1.2 × 10^{12} vg of AAV-EGFP at individually defined disease onset.

- A Kaplan–Meier survival curves after birth of untreated ALS mice (n = 15 mice, 158.4 ± 1.9 days, mean ± SEM) and AAV-EGFP-treated ALS mice (n = 14 mice, 162.1 ± 2.5 days). n.s., not significant by log-rank test. NT, not treated.
- B Kaplan–Meier survival curves after onset in untreated ALS mice (n = 15 mice, 57.3 ± 2.4 days, mean ± SEM) and AAV-EGFP-treated ALS mice (n = 14 mice, 59.8 ± 3.0 days). n.s., not significant by log-rank test.
- C Ages of onset for untreated (n = 15 mice) and AAV-EGFP-treated ALS mice (n = 14 mice). Mean ages of onset were indicated by horizontal bars. n.s., not significant by Student's t-test.
- D Forelimb grip strength of ALS mice at onset in untreated (n = 15 mice) and AAV-EGFP-treated groups (n = 14 mice). Values are means ± SEM. n.s., not significant by Student's t-test.
- E The difference in cycle threshold (ΔC_t) between the human *SOD1-G93A* transgene and the reference mouse *apob* gene. To calculate the human transgene level, the ΔC_t value of *hSOD1* was subtracted from the ΔC_t value of *apob* (ALS-NT, n = 15 mice; ALS-AAV-EGFP, n = 14 mice).

© 2017 The Authors

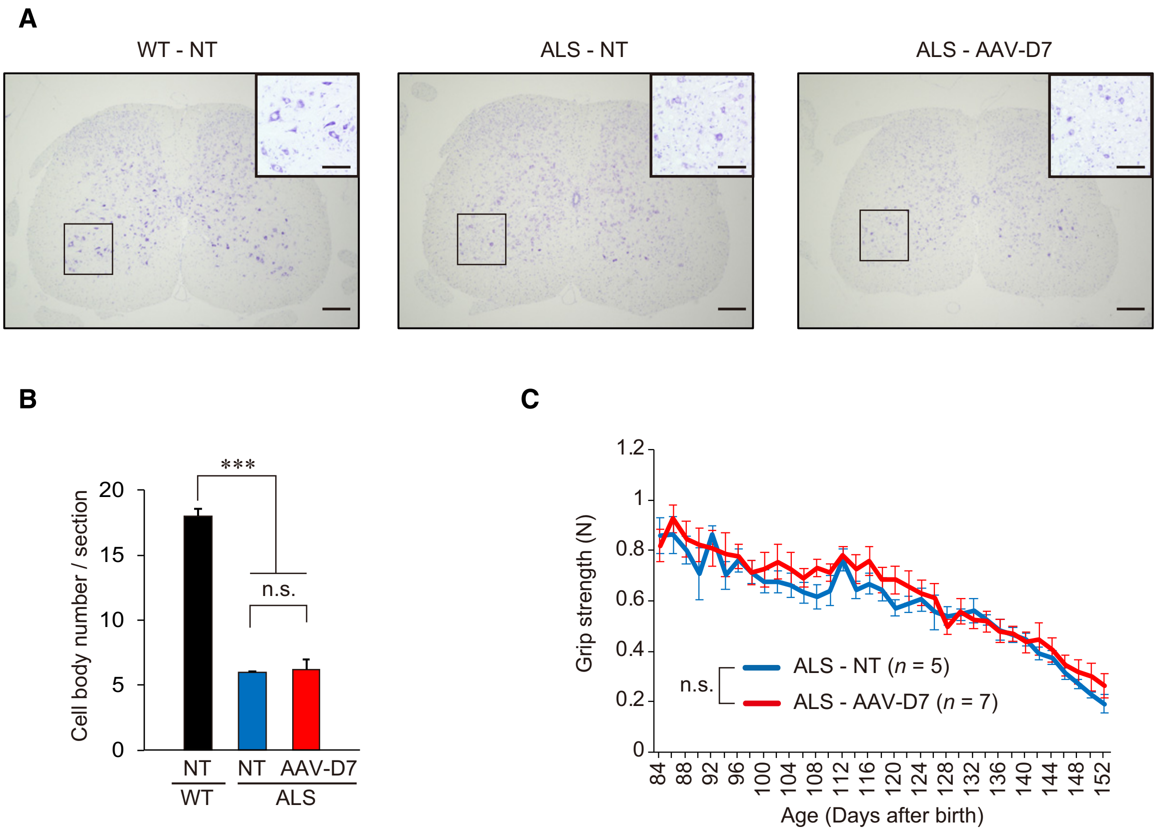

Figure EV4. Treatment with AAV-D7 at individually defined disease onset shows no obvious effects on motor neuron survival or grip strength in ALS mice.
WT or ALS mice were treated or not with 1.2×10^{12} vg of AAV-D7 at individually defined disease onset.

A Nissl staining of the L4-L5 spinal segment at P150. Scale bars, 200 μm. Insets show higher magnification of the ventral horn in the boxed regions (scale bars, 100 μm). NT, not treated.

B Quantification of surviving motor neurons at P150 (WT-NT, $n = 3$ mice; ALS-NT, $n = 4$ mice; ALS-AAV-D7, $n = 4$ mice). Values are means ± SEM. ***$P < 0.0001$ by one-way ANOVA with Bonferroni's *post hoc* test. n.s., not significant.

C Forelimb grip strength analysis at the indicated ages for ALS-NT ($n = 5$ mice) and ALS-AAV-D7 ($n = 7$ mice). Values are means ± SEM. n.s., not significant by Student's *t*-test.

 © 2017 The Authors
