# Supplementary figures and images for: DOK7 gene therapy enhances motor activity and life span in ALS model mice
Source: EMBO Mol Med. 2017 May 10;9(7):880–9. doi: 10.15252/emmm.201607298 (PMC5494517; doi:10.15252/emmm.201607298)

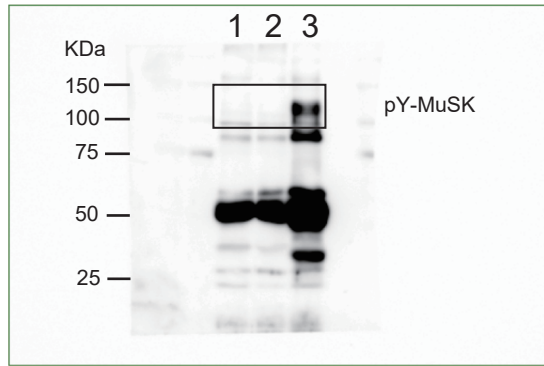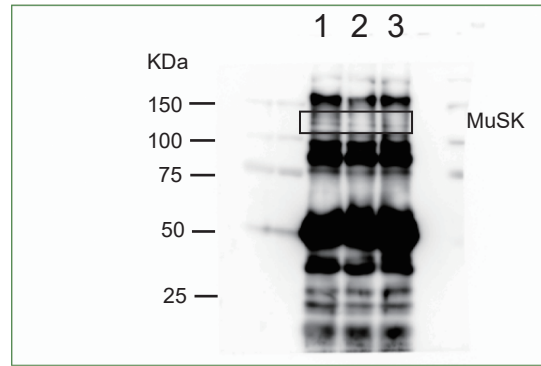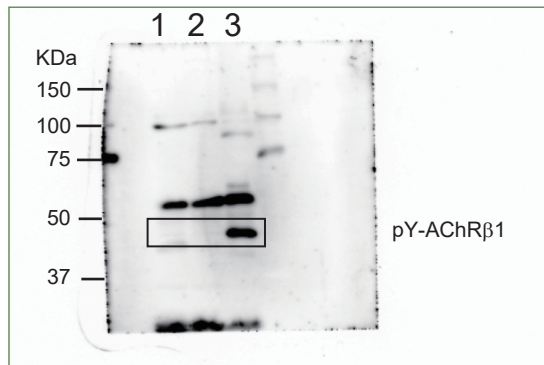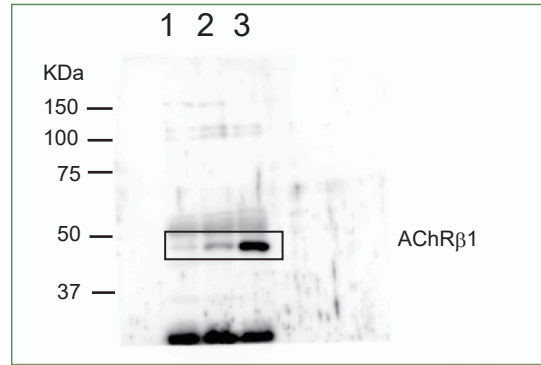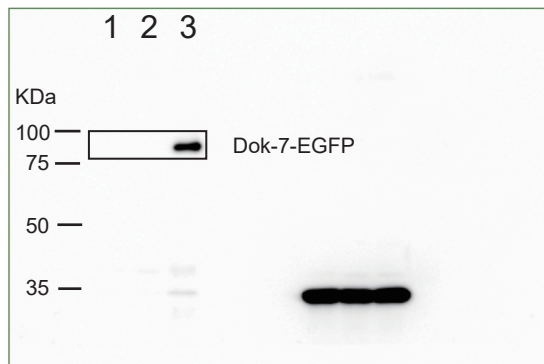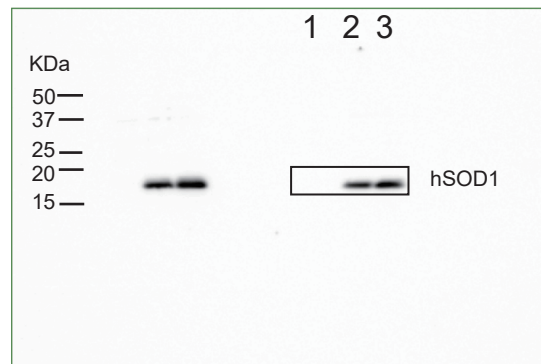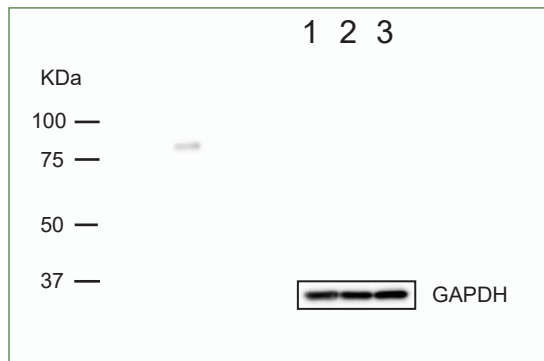

**Samples:**

1 WT - NT

2 ALS - NT

3 ALS - AAV-D7

Supplement: Supplementary file 4 — Source Data for Figure 1 [file EMMM-9-880-s003.pdf]
